# The subtle balance of weak supramolecular interactions: The hierarchy of halogen and hydrogen bonds in haloanilinium and halopyridinium salts

**DOI:** 10.3762/bjoc.6.4

**Published:** 2010-01-15

**Authors:** Kari Raatikainen, Massimo Cametti, Kari Rissanen

**Affiliations:** 1 Nanoscience Center, Department of Chemistry, University of Jyväskylä, P.O. Box 35, 40014 Jyväskylä, Finland

**Keywords:** crystal engineering, halogen bonding, hydrogen bonding, supramolecular chemistry, weak interactions

## Abstract

The series of haloanilinium and halopyridinium salts: 4-IPhNH_3_Cl (**1**), 4-IPhNH_3_Br (**5**), 4-IPhNH_3_H_2_PO_4_ (**6**), 4-ClPhNH_3_H_2_PO_4_ (**8**), 3-IPyBnCl (**9**), 3-IPyHCl (**10**) and 3-IPyH-5NIPA (3-iodopyridinium 5-nitroisophthalate, **13**), where hydrogen or/and halogen bonding represents the most relevant non-covalent interactions, has been prepared and characterized by single crystal X-ray diffraction. This series was further complemented by extracting some relevant crystal structures: 4-BrPhNH_3_Cl (**2**, CCDC ref. code TAWRAL), 4-ClPhNH_3_Cl (**3**, CURGOL), 4-FPhNH_3_Cl (**4**, ANLCLA), 4-BrPhNH_3_H_2_PO_4_, (**7**, UGISEI), 3-BrPyHCl, (**11**, CIHBAX) and 3-ClPyHCl, (**12**, VOQMUJ) from Cambridge Structural Database for sake of comparison. Based on the X-ray data it was possible to highlight the balance between non-covalent forces acting in these systems, where the relative strength of the halogen bonding C–X···A^−^ (X = I, Br or Cl) and the ratio between the halogen and hydrogen bonds [C–X···A^−^ : D–H···A^−^] varied across the series.

## Introduction

Non-covalent interaction, such as hydrogen bonding and metal coordination represent the basic set of tools for the construction of elaborate architectures in the supramolecular chemistry of organic or metal-organic compounds [1]. In the past few years, there has been a growing interest towards the development of new types of intermolecular interactions. In particular, halogen bonding has attracted significant attention and it is considered nowadays as a promising instrument in supramolecular chemistry [2]. Halogen bonding (XB) is the non-covalent interaction involving halogen atoms as electrophilic species [3]. The first reports of these interactions, only later classified as halogen bonds, date back to the late 1960’s [4]. In the following years, several X-ray studies demonstrated the existence of the short interaction distance between the halogen atom and a nucleophilic atom in a number of crystal structures [5–6]. In 1996 Allen and co-workers [7] did an extensive statistical analysis of all of the crystal structures in the Cambridge Structural Database (CSD) for carbon-bound halogen atoms (C–X where X = F, Cl, Br or I) and nucleophilic atoms (S, O or N, in their various hybridization states). The analysis was based on intermolecular contact distances shorter than 1.26 times the sum of the van der Waals (VDW) radii of the two interacting atoms. The analysis showed that the intermolecular contacts between halogen (Cl, Br, and I but not F) atoms and nucleophilic (O and N) atoms manifest a highly directional, attractive interaction leading to contact distances clearly shorter than the sum of VDW radii [7]. They also concluded that the attractive nature of the interaction is mainly due to electrostatic effects, but polarization, charge-transfer, and dispersion contributions all play an important role, more recently confirmed also by theoretical and experimental studies [8–10].

Interactions between halogens and nucleophilic atoms were generally considered to be too weak to be used in crystal engineering, until the late ’90s when G. Resnati and P. Metrangolo [11–16] made a major breakthrough in the field by exploring the use of perfluorocarbon (PFC) iodides and aliphatic amines in the formation of strong halogen–nucleophile interactions, from then systematically called “halogen bonding”. In these systems, the C_PFC_–I···N contact distances are usually around 2.8 Å corresponding to ca. 20% reduction of the sum of standard VDW radii of nitrogen (1.55 Å) and iodine (1.98 Å) [17]. The strong interaction between the highly polarized iodine and the nitrogen atom, manifested by the remarkably short interaction distance, has been shown to overcome the low affinity between hydro- and perfluorinated carbon molecules by effectively forming stable high melting co-crystals. Since then, this novel interaction has become a common tool in supramolecular chemistry, especially in crystal engineering [18–19], and lately it has widely and successfully applied in other fields of material science, such as in supramolecular separations, liquid crystals, organic semiconductors and paramagnetic materials technologies [20–21]. Recently, the important role of XBs in biological systems and its potential in drug development has also been recognized [22].

The halogen bond (XB), whose terminology emphasizes the similarity with hydrogen bonding [23] can be schematically described by Y–X···A, where X is the XB donor atom (Lewis acid, electrophilic) and A is the XB acceptor atom (Lewis base, nucleophilic) [20]. According to this definition, halogen bonding covers a vast family of non-covalent interactions, and a very wide range of interaction energies [20]. Concurrently with the development of practical applications and experimental studies on halogen bonding systems, theoretical and conceptual aspects of halogen bonding have been scrutinized in detail. Theoretical studies [24–25] of halogen bonding show that the electron density is anisotropically distributed around the covalently bound halogen atom. A region of a positive electrostatic potential is formed at the surface of the halogen atom, localized along the extension of the Y–X···A covalent bond. The existence and magnitude of this positive region, known as σ-hole [25], depends on the polarizability of the halogen atom, and by no surprise the interaction energy is found to increase in by the order Cl < Br < I [26], following the polarizability of halogen atom. The hybridization of the C–X carbon atom on the XB donor molecule has also an effect on the strength and directionality of the halogen bond. The order C(sp^3^) < C(sp^2^) < C(sp) is generally followed [24–26] and for example haloalkynes are found to be particularly good halogen bond donors [27–28]. As seen in PFC compounds, electron withdrawing moieties present on the Y group favor the interaction. For this reason haloarenes where the aromatic ring has electron withdrawing substituents e.g. fluorines [11–16,18–19] are also excellent halogen bond donors. Iodonitrobenzene derivatives represent a less explored type of haloarenes [29–30]. In these XB systems, secondary C–I···O_2_N_Ar_ halogen bonds (distances 13% shorter than the sum of standard VDW radii [17]) have been observed for iodonitrobenzenes themselves [31–32] or in co-crystals of iodo- and nitrobenzenes [29–30]. In our recent studies [33], we have shown that 1-iodo-3,5-dinitrobenzene forms surprisingly strong C–I···N halogen bonds (23% shorter than the sum of standard VDW radii [17]) with 1,4-diazabicyclo[2.2.2]octane (DABCO).

One of the main challenges in supramolecular chemistry and crystal engineering is to identify the hierarchies of non-covalent interactions in order to develop efficient synthetic strategies for attaining advanced supramolecular systems [1]. The structure of a supramolecular assembly in crystalline solids generally results from the balance of all intermolecular interactions in the crystal, which results from maximizing the attractive interactions and minimizing the repulsive ones, generally affording the densest of packing. When two major interactions, such as hydrogen bonding (HB) and halogen bonding (XB), are simultaneously present in a system, it is not always straightforward to predict which one of them is going to determine the overall crystal architecture. In some cases, the strength of the halogen bond interactions allows them to overrule hydrogen bonds in the hierarchy of intermolecular interactions [15,34]. Recently it has been proposed [35–36] that the hierarchy of intermolecular non-covalent interactions carefully balancing hydrogen- and halogen bonding can be affected and thus applied in rational design of supramolecular entities and crystal structures.

**Scheme 1:**
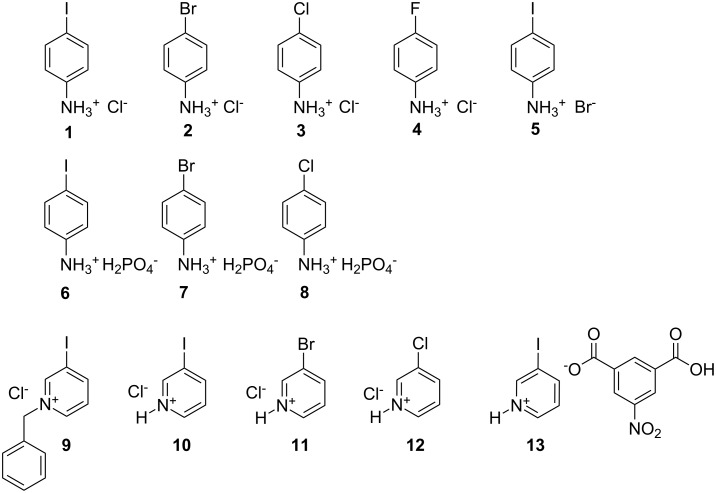
The chemical structures of the salts **1–13**.

In this paper, we describe a number of simple haloanilinium and halopyridinium salt structures which clearly show how the balance of intermolecular interactions such as HB and XB can determine the supramolecular architectures found in the solid state (Scheme 1).

The detailed study of the seven new crystal structures, namely anilinium salts 4-IPhNH_3_Cl (**1**), 4-IPhNH_3_Br (**5**), 4-IPhNH_3_H_2_PO_4_ (**6**), 4-ClPhNH_3_H_2_PO_4_ (**8**) and corresponding pyridinium salts 3-IPyBnCl (**9**), 3-IPyHCl (**10**) and 3-IPyH-5NIPA (3-iodopyridinium 5-nitroisophthalate, **13**), complemented by the comparison with corresponding structures found in the literature, reveals the subtle balance between HB and XB in these salts. The structures of salts 4-BrPhNH_3_Cl (**2**, CCDC ref. code TAWRAL) [37], 4-ClPhNH_3_Cl (**3**, CURGOL) [38], 4-FPhNH_3_Cl (**4**, ANLCLA) [39], 4-BrPhNH_3_H_2_PO_4_, (**7**, UGISEI) [40], 3-BrPyHCl, (**11**, CIHBAX) [41] and 3-ClPyHCl, (**12**, VOQMUJ) [42] were extracted from the CSD [43] in order to obtain the full homogeneous series.

## Results and Discussion

In addition to the exact measurement of C–X···A contact distances, we also calculated the relative XB distances **R** (Equation 1), following the definition of Lommerse et al., [7] where standard VDW radii of interacting atoms were taken into account to bring interaction distances into the standardized scale.

[1]
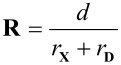


Here, *d* is X···D distance and *r*_X_ and *r*_D_ (or *r*_ion_) are standard VDW radii of the involved atoms (or ions) (*r*_Cl_- = 1.81 Å, *r*_Cl_ = 1.75 Å, *r*_Br_ = 1.85, *r*_Br_- = 1.96 Å, *r*_I_ = 1.98, *r*_O_ = 1.52) [17,44]. In addition to the relative XB distances **R**, the ratio of the most relevant interactions, that are the charge assisted hydrogen and halogen bonds, were taken into the consideration. The ratio (D^+^–H···) : (Y–I···), namely hydrogen bonding and halogen bonding, donor sites in haloanilinium halides is 3 : 1, whereas in H_2_PO_4_ salts it is 5 : 1. In halopyridinium salts corresponding ratio of donor sites vary from a solely halogen bonding (0 : 1) system to a 2 : 1 ratio in **13**.

### Halogen and hydrogen bonding in 4-IPhNH_3_Cl (1), 4-BrPhNH_3_Cl (2), 4-ClPhNH_3_Cl (3) and 4-FPhNH_3_Cl (4)

The first four structures (**1–4**) form a series of haloanilinium chlorides (Scheme 1) carefully chosen to probe the effect of the halogen substituent on the balance of HB and XB in these systems.

**Figure 1:**
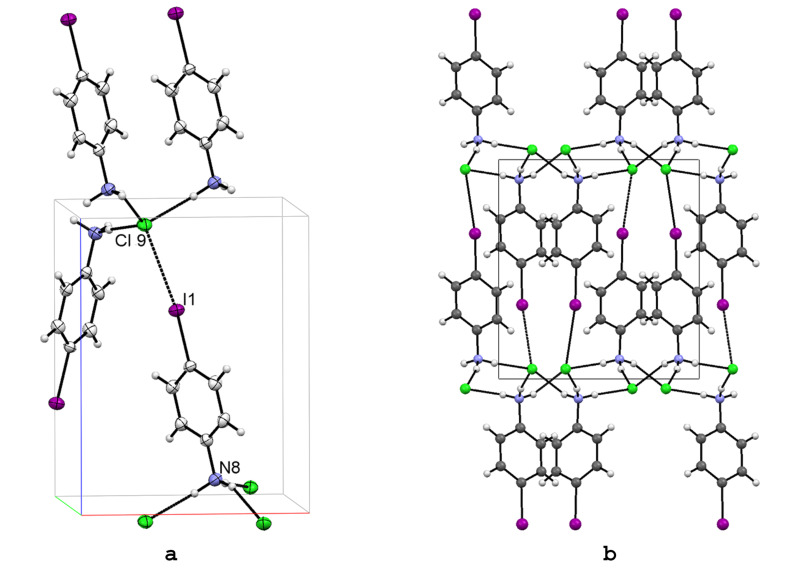
X-ray structure of 4-IPhNH_3_Cl (**1**) with numbering for selected atoms (a) and the packing scheme viewed down the *a* axis (b). Thermal ellipsoids are shown at the 50% probability level. Hydrogen and halogen bonds are shown in dotted lines.

X-ray-quality crystals of **1** were obtained by crystallization of 4-iodoaniline from ethanol–HCl solution (Figure 1a). The halogen bond I···Cl^−^ is about 10% shorter than the sum of standard VDW radii of the interacting atoms (3.79 Å) [17,44], definitely weaker than in the classical PFC-I···N systems [11–16]. The crystal packing reveals a pattern of complementary donor and acceptor sites for three N^+^–H···Cl^−^ hydrogen bonds, which in addition to one I···Cl^−^ mentioned above, creates a distorted tetrahedral coordination sphere around the Cl^−^ anion (Figure 1a). The N^+^–H···Cl^−^ hydrogen bonds are situated on the *a,b*-plane forming 2D hexagonal network (Figure 1b). The iodobenzene moieties, perpendicular to the hydrogen bond network, are segregated between these HB layers, with the halogen bonding acting as an anchor to the fourth coordination site of the Cl anion, to further stabilize the structure in direction of the *c* axis.

**Figure 2:**
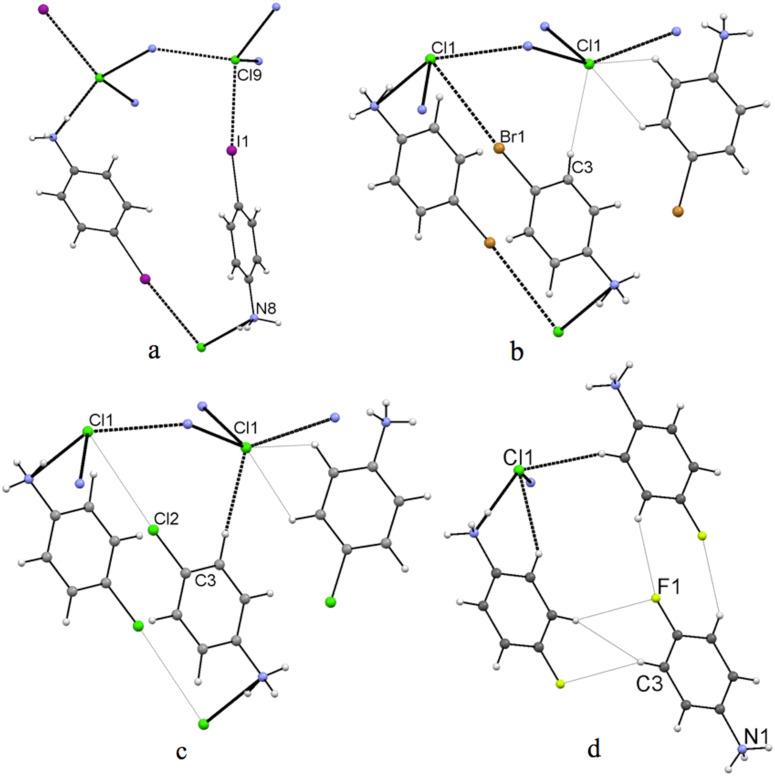
Interaction contacts in 4-IPhNH_3_Cl (**1**; a), 4-BrPhNH_3_Cl (**2**; b), 4-ClPhNH_3_Cl (**3**; c) and 4-FPhNH_3_Cl (**4**; d). Dotted lines represent the hydrogen and halogen interactions, where the shorter (stronger) contact distances are shown in bold lines and the longer (weaker) with narrow lines.

To gain more information about the effect of the halogen (X) identity on C–X···Cl^−^ halogen bonding distances, the structure of 4-IPhNH_3_Cl (**1**) was compared with a series of *p*-substituted bromo- (**2**), chloro- (**3**), and fluoroanilinium chlorides (**4**) published previously. Based on these previous experimental and theoretical studies [24–26], halogen bond strength was expected to vary from a clearly non-existent F···Cl^−^ interaction to most attractive I···Cl^−^ interaction. Comparing these analogous structures, where instead the charge assisted hydrogen bond network is kept constant, the relative strength and role of halogen bond in crystal architecture can be evaluated. In this respect, the variation of the size of the VDW radii of the halogen atom was considered to have a minor effect in the present context. In all these crystals, the structurally similar charge-assisted hydrogen bond network is the main structural feature, which determines the overall orientation of the molecules [see packing of 4-IPhNH_3_Cl (**1**) in Figure 1b]. Halogen bonding is evident only in the structure of 4-IPhNH_3_Cl (**1**; Figure 2a), but weak halogen bonding Br···Cl^−^ is observed in 4-BrPhNH_3_Cl (**2**, TAWRAL [39]; Figure 2b) as well.

In 4-ClPhNH_3_Cl (**3**, CURGOL [39]; Figure 2c), distance Cl···Cl^−^ is slightly longer than Br···Cl^−^ and the sum of VDW radii [17,44]. However, the structures of 4-BrPhNH_3_Cl (**2**) and 4-ClPhNH_3_Cl (**3**) are isomorphous. The measured X···Cl^−^ distances, angles and other pertinent structural data are given in Table 1. At variance with the other members of the series, the structure of fluoro-substituted anilinium chloride **4** [39] is completely different (Figure 2d) and does not show similar hydrogen bonding and no halogen bonding and thus it was excluded from Table 1.

**Table 1:** Relevant C–X, hydrogen bond and halogen bond lengths and angles in **1**–**3**.

	C–X [Å] [X]	X···Cl^−^ [Å] [Cl^−^] [**R**]*	C–X···Cl^−^ [°]	Cl^−^···H–N_a_ [Å]	N_a_···Cl^−^···N_b_

4-IPhNH_3_Cl, **1**	2.102 [I1]	3.405 [Cl9] [0.90]	169.8°	3.049	108.8°
				3.092	117.1°
				3.103	110.6°
4-BrPhNH_3_Cl, **2** (TAWRAL [37])	1.892 [Br1]	3.587 [Cl1] [0.98]	165.9°	3.135	87.3°
				3.161	139.2°
				3.143	106.8°
4-ClPhNH_3_Cl, **3** (CURGOL [38])	1.741 [Cl2]	3.635 [Cl1] [1.02]	166.6°	3.135	85.8°
				3.157	138.5°
				3.115	106.9°

^* ^**R** = *d*/(*r*_X_ + *r*_D_), see Equation 1.

This difference can be explained by the fact that, instead, the fluorine substituent forms weak F···H hydrogen bonds with aryl hydrogens (Figure 2d). It is also interesting to note that the intermolecular interaction pattern in 4-IPhNH_3_Cl (**1**) differs from the isomorphic chloro- and bromo-derivates **2** and **3** and is explained by the existing, though quite weak, I···Cl^−^ halogen bond.

Detailed inspection of the structures **1**–**4** revealed that non-covalent tetrahedral coordination of Cl^−^ by three charge-assisted hydrogen bonds and one halogen bond exists only in structure of *p*-iodo salt **1** (Figure 2a), resulting in a more linear C–I···Cl^−^ interaction angle, which is consistent with the shorter XB distance. Also the HB distances are shorter. When compared to the *p*-bromo and *p*-chloro structures **2** and **3** (Figure 2b and Figure 2c), the weaker halogen bonding tendency reverts the orientation of the benzene moiety to a closed dimer motif. As a conclusion, the C–Br···Cl^−^ and C–Cl···Cl^−^ interactions in **2** and **3** are not comparable to the halogen bond in **1**, but can be considered as intermediate structures between the truly halogen bonded **1** and the only hydrogen bonded **4**. When the polarizability of the halogen atom is increased (I > Br > Cl > F), thus increasing the effect of the halogen bond, the changed balance of the intermolecular interactions will influence the spatial organization of the adjacent molecules leading to a different crystal architecture. The strong charge-assisted hydrogen bonding clearly overrules the weaker halogen bonding and is the major cause for the crystal packing.

### Halogen and hydrogen bonding in 4-IPhNH_3_Br (5)

**Figure 3:**
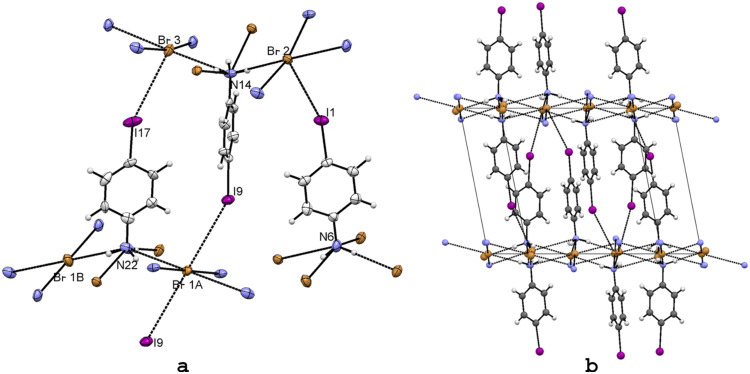
X-ray structure of 4-IPhNH_3_Br (**5**) with selected numbering scheme (a) and the packing scheme viewed down the *a* axis (b). Thermal ellipsoids are drawn at the 50% probability level. Hydrogen and halogen bonds are shown in dotted lines.

Exchanging the chlorine counter anion for bromine was expected to give weaker halogen bond interactions due to the lower nucleophilicity of the bromine anion, but also hydrogen bonding distances and coordination were expected to be different. Crystallization from ethanol–HBr solution resulted in crystals of **5** in which the asymmetric unit contains three molecules of *p*-iodoanilinium bromide (Figure 3a). The main structural feature of **5** is, surprisingly, the very similar overall HB motif (Figure 3b) with the one in 4-IPhNH_3_Cl (**1**; Figure 1a), despite the clearly different coordination around the Br anion (Figure 3a). The 4-IPhNH_3_Br (**5**) displays quite long I···Br^−^ XB distances, shown in Table 2, being only slightly shorter than the sum of VDW. The weaker interactions, i.e. the longer I···Br^−^ distances, manifest the lower nucleophilicity of the Br anion. Even though the I···Br^−^ distances are relatively long, the quite linear C-I···Br^−^ bond angle supports the presence of weak XB interaction, clearly weaker that in the corresponding anilinium chloride **1**.

**Table 2:** Relevant covalent bond, hydrogen bond and halogen bond lengths and angles in **5**.

	C–I [Å] [I]	X···Br^−^ [Å] [Br^−^] [**R**]*	C–X···Br^−^	Br^−^···H–N_a_ [Br···N] [Å]	N_b_···Br^−^···N_a_

4-IPhNH_3_Br, **5**	2.102 [I1]	3.704 [Br2] [0.94]	158.4°	3.265 [Br2···N14]	87.3° [N22··Br2···N14]
				3.408 [Br2···N22]	92.3° [N6···Br2···N22]
				3.347 [Br2···N6]	90.8° [N14···Br2···N6]
				3.441 [Br2···N14]	89.8° [N14···Br2···N14]
	2.083 [I17]	3.834 [Br3] [0.97]	154.1°	3.257 [Br3···N6]	81.7° [N22···Br3···N6]
				3.294 [Br3···N22]	96.0° [N14···Br3···N22]
				3.333 [Br3···N14]	93.6° [N6···Br3···N14]
				3.269 [Br3···N6]	88.5° [N6···Br3···N6]
	2.087 [I9]	3.893 [Br1A] [0.99]	148.5°	3.310 [Br1A···N22]	86.2° [N14···Br1A···N22]
				3.430 [Br1A···N14]	93.8° [N22···Br1A···N14]
		[Br1B]		3.219 [Br1B···N22]	82.6° [N6···Br1B···N22]
				3.269 [Br1B···N6]	97.4° [N22···Br1B···N6]

^* ^**R** = *d*/(*r*_X_ + *r*_D_), see Equation 1.

### Halogen and hydrogen bonding in 4-IPhNH_3_H_2_PO_4_ (6), 4-BrPhNH_3_H_2_PO_4_ (7) and 4-ClPhNH_3_H_2_PO_4_ (8)

**Figure 4:**
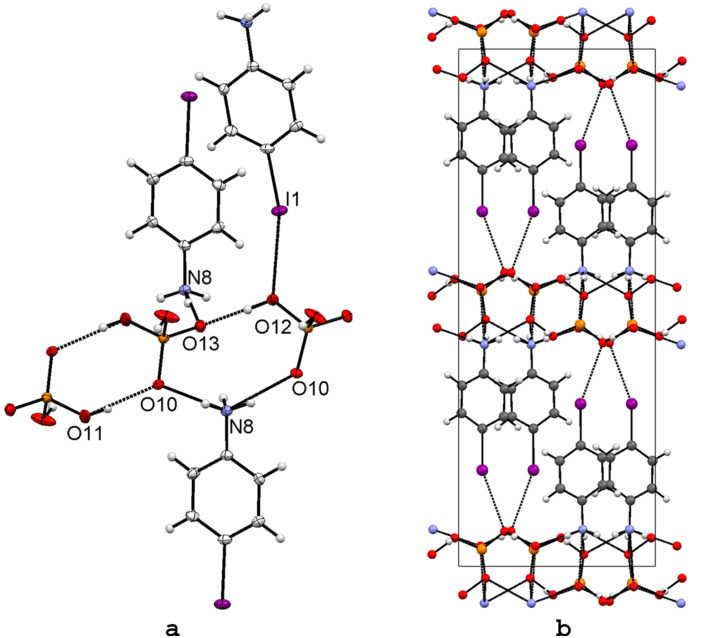
X-ray structure of 4-IPhNH_3_H_2_PO_4_ (**6**) with selected numbering scheme of the asymmetric unit and the packing scheme viewed down the *a* axis (b). Thermal ellipsoids are drawn at the 50% probability level. Hydrogen and halogen bonds are shown in dotted lines.

The balance between the halogen bonding and hydrogen bonding in anilinium salts can be also modulated by the exchange of the spherical halide anions with tetrahedral anions such as dihydrogenphosphate. In addition, H_2_PO_4_^−^ ion offers two OH groups providing two additional hydrogen bond donor sites differing from the corresponding anilinium halides, thus the interaction type ratio (D^+^–H···) : (C–I···) in **6** is 5 : 1. As the dihydrogenphosphate anion is a stronger hydrogen bond acceptor that the halide anions (Cl^−^, Br^−^ or I^−^), it was interesting to study whether the weak halogen bonding observed in the anilinium halide salts **1** and **2** would be completely overruled by the dihydrogenphosphate anion or not. Crystals of 4-IPhNH_3_H_2_PO_4_ (**6**) were obtained from a methanol–phosphoric acid solution of 4-iodoaniline by slow evaporation. The asymmetric unit of **6** is depicted in Figure 4a. The hydrogen bonding pattern consists of three N–H···O and two O–H···O interactions, as expected. Hence the H_2_PO_4_ anions and H_3_N^+^ moieties are H-bonded together forming a 2D layer of strong hydrogen bonds. The layers are perpendicular to the crystallographic *c* axis and the spacing between the layers is about 13 Å. Aromatic moieties are segregated between these layers, thus the overall crystal packing (Figure 4b) is very similar to the haloanilinium halides (Figure 1 and Figure 3). Unexpectedly, a quite strong I1···O12 halogen bond with **R** = 0.93 is formed between the iodine atoms and one of the O atoms in the dihydrogenphosphate anion. The XB angle C–I1···O12 is ~ 160°, which is consistent with the halogen bonds seen in 4-IPhNH_3_Br (**5**), 4-BrPhNH_3_Cl (**2**) and 4-ClPhNH_3_Cl (**3**) structures.

Hydrogen bonding clearly dominates the crystal packing of 4-IPhNH_3_H_2_PO_4_ (**6**). Yet the most nucleophilic oxygen atom in the dihydrogenphosphate anion acts as a halogen bond acceptor towards the moderately polarized iodine atom. The relative strength of the halogen bonding can be tuned by changing the polarizability of the halogen atom as manifested by the anilinium salts discussed above. Thus, substitution of the iodine atom for bromine, as in 4-BrPhNH_3_H_2_PO_4_ (**7**, UGISEI [40]) was expected to show longer XB interaction distances due to the lower polarizability of the bromine atom [24–26]. In **7**, the XB distance Br···O is 3.348 Å, with **R** = 0.99 (Table 3), thus reflecting the weaker or nearly non-existent interaction. In spite of the slight differences in the halogen bonding interactions the crystal structures of **6** and **7** are isomorphic. This feature indicates that the weak halogen bonding observed in **6** is not able to overrule the strong hydrogen bonding induced by the dihydrogenphosphate, as in the case of the chloride (a weaker hydrogen bonding donor) in the structure of **1**. To prove that indeed the **R** = 0.99 in **7** does not represent halogen bonding interactions, we crystallized *p*-chloroaniline from ethanol–phosphoric acid solution to get the crystal structure of the corresponding 4-ClPhNH_3_H_2_PO_4_ (**8**). As expected, the **R** = 1.00 in **8** and the structure is isomorphic with **6** and **7**. Table 3 shows that due to the strong and governing hydrogen bonding by the dihydrogenphosphate the X···O distances and C–X···O contact angles do not show the trend observed in the haloanilinium chlorides (**1**–**3**).

**Table 3:** Relevant covalent bond, hydrogen bond and halogen bond lengths and angles in **6**–**8**.

	C–X [Å] [X]	X···O [Å] [O] [**R**]*	C–X···O	D–H···O [D]	D–H···O [O]

4-IPhNH_3_H_2_PO_4_, **6**	1.892 [I1]	3.262 [O12] [0.93]	159.5°	2.930 Å [N8]	165.8° [O10]
				2.860 Å [N8]	173.9° [O10]
				2.707 Å [N8]	173.7° [O13]
				2.598 Å [O11]	155.6° [O10]
				2.533 Å [O12]	160.3° [O13]
4-BrPhNH_3_H_2_PO_4_, **7 **(UGISEI, [40])	1.902 [Br1]	3.348 [O1] [0.99]	157.2°	2.951 Å	
				2.873 Å	
				2.701 Å	
				2.582 Å	
				2.540 Å	
4-ClPhNH_3_H_2_PO_4_, **8**	1.742 [Cl1]	3.260 [O2] [1.00]	156.9°	2.920 Å [N8]	164.7° [O10]
				2.844 Å [N8]	175.4° [O10]
				2.678 Å [N8]	174.7° [O13]
				2.590 Å [O11]	161.4° [O10]
				2.534 Å [O12]	158.8° [O13]

^* ^**R** = *d*/(*r*_X_ + *r*_D_), see Equation 1. D represents the hydrogen bond donor atom.

### Halogen bonding in 3-IPyBnCl (9)

**Figure 5:**
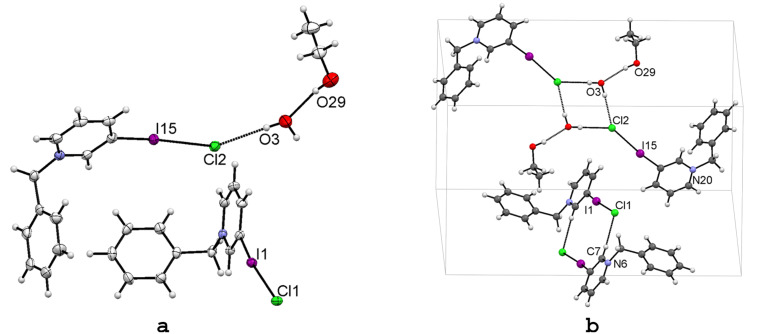
X-ray structure of 3-IPyBnCl (**9**) with the selected numbering scheme of the asymmetric unit (a) and selected packing scheme viewed down the *a* axis (b). Thermal ellipsoids are drawn at the 50% probability level. Hydrogen and halogen bonds are shown in dotted lines.

One additional way to polarize the halogen atom is to attach it into a charged aromatic ring, as in the pyridinium moiety where the positive charge is delocalized over the aromatic ring inducing a stronger polarizing effect to the halogen substituent. By no surprise, short halogen bond interactions are characteristic in halopyridinium salts [45–47]. Depending on the structure of the pyridinium moiety, namely protonated N^+^–H or *N*-alkylated N^+^–R, the hydrogen bonding interactions between the molecular components can be influenced. The protonated pyridinium is a very strong hydrogen bond donor whereas the *N*-alkylated pyridinium is not. Thus the ratio of [N^+^–H···] and [C–I···], HB and XB, donor sites is 0 : 1 [N^+^–R] or 1 : 1 [N^+^–H]. To override the hydrogen bond contribution we first focused our attention on *N*-benzylpyridinium salts, which should completely suppress the strong hydrogen bond interactions and give space to strong XB interaction instead if an iodine substituent would sit on the aromatic ring. Therefore, we prepared *N*-benzyl-3-iodopyridinium chloride (**9**) by nucleophilic substitution reaction of 3-iodopyridine with (chloromethyl)benzene (the synthesic details will be reported elsewhere). Slow evaporation of a moist ethanol solution gave an X-ray-quality crystal of **9**. The asymmetric unit contains two molecules of *N*-benzyl-3-iodopyridinium chloride, a water molecule and an ethanol solvent molecule (Figure 5a). The electron withdrawing effect of *N*-benzylpyridinium cation gives rise to short halogen bonds, where the **R** = 0.83 and **R** = 0.85 for I1···Cl1 and I15···Cl2, respectively (Table 4). The short halogen bond distances are consistent with the linearity of C–I1···Cl1 and C–I15···Cl2 angles [174.1(1)° and 174.6(1)°, respectively].

**Table 4:** Relevant covalent bond, hydrogen bond and halogen bond lengths and angles in **9**–**12**.

	C–X [Å] [X]	X···Cl^−^ [Å] [Cl^−^] [**R**]*	C–X···Cl^−^	Cl^−^···H–N [Å]	N–H···Cl^−^

3-IPyBnCl, **9**	2.101 [I1]	3.151 [Cl1] [0.83]	174.1°	–	–
	2.099 [I15]	3.223 [Cl2] [0.85]	174.6°	–	–
3-IPyHCl, **10**	2.096 [I1]	3.189 [Cl1] [0.84]	174.3°	3.035	163.0°
	2.114 [I8]	3.170 [Cl4] [0.84]	179.7°	3.058	146.0°
	2.105 [I15]	3.141 [Cl1] [0.83]	177.3°	3.044	149.4°
	2.096 [I22]	3.227 [Cl4] [0.85]	173.8°	3.024	164.9°
3-BrPyHCl, **11** (CIHBAX [41])	1.890 [Br1]	3.359 [Cl1] [0.89]	162.2°	2.995	152.9°
3-ClPyHCl, **12** (VOQMUJ [42])	1.727 [Cl1]	3.479 [Cl2] [0.92]	156.1°	2.993	169.4°

^* ^**R** = *d*/(*r*_X_ + *r*_D_), see Equation 1.

Since the alkylation on the N atom prevents any hydrogen bond interactions with pyridine, the packing is predominantly driven by halogen bonds. As shown in Figure 5b, two independent and structurally different interaction motifs are present in the crystal lattice. The first is a dimeric motif with two symmetry equivalent *N*-benzyl-3-iodopyridinium chloride moieties coordinating through XB and weak HB, C–I1···Cl1^−^···H–C7, interactions (Figure 5b, below). In the other motif (Figure 5b, top), the chlorine anion coordinates the pyridinium ions and water with XB, C–I15···Cl2^−^, and HB, Cl2^−^···H–O3, interactions. The water molecule [O3] bridges the chloride [Cl2] anions creating a parallelogram-shaped hydrogen bonded dimer. In addition, O3 forms another hydrogen bond with a solvent ethanol molecule. Interestingly, of the two independent halogen bonds (C–I1···Cl1 and C–I15···Cl2), the latter displays a slightly longer contact distance (Table 4), and this is due to the involvement the chloride atom in a second interaction, a hydrogen bond with a water molecule, which consequently weakens its I15···Cl2 interaction.

### Halogen and hydrogen bonding in 3-IPyHCl (10) 3-BrPyHCl (11) and 3-ClPyHCl (12)

**Figure 6:**
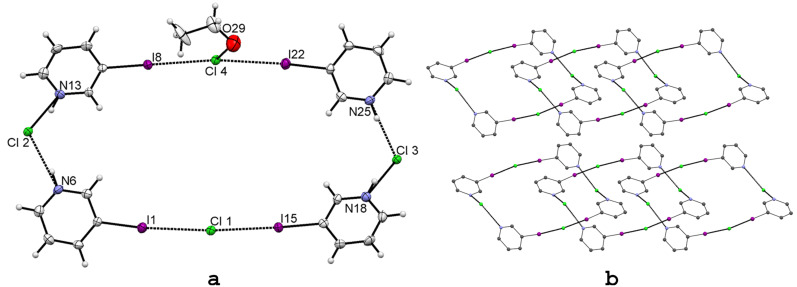
X-ray structure of 3-IPyHCl (**10**) with the selected numbering scheme of the asymmetric unit (a) and packing scheme viewed down the crystallographic *c* axis (b). Thermal ellipsoids are drawn at the 50% probability level. Hydrogen and halogen bonds are shown in dotted lines.

As in all above studied salts **1**–**9**, similar type X···Cl interactions are also possible in 3-iodo-, 3-bromo- and 3-chloropyridinium chlorides (**10**–**12**). However, as the pyridinium cation is obtained by protonation of the pyridine nitrogen, the very strong hydrogen bond donor [N^+^–H···] moiety is envisaged to disrupt or severely hinder the strong halogen bonding interactions manifested in the non-HB pyridinium salt **9**. Slow diffusion of ethyl acetate into the ethanol solution of 3-iodopyridinium chloride gave an X-ray-quality crystal of **10**. The asymmetric unit contains four molecules of 3-iodopyridinium chloride and one molecule solvent ethanol (Figure 6a). As in 3-IPyBnCl (**9**), the electron withdrawing effect of pyridinium cation in **10** gives rise to four short C–I···Cl^−^ halogen bonds, from which the shortest, in I15···Cl1, **R** = 0.83, is the same as in the non-HB salt **9**. The XB distances and angles are very similar as in **9** (Table 4), thus halogen bonding is not weakened even the presence of strong charge-assisted hydrogen bond, N^+^–H···Cl^−^. This can be explained by the segregation of the XB and HB interactions. In fact, two of the four chloride anions [Cl1 and Cl4] are engaged only with the halogen bonding (one of them in addition of O29–H···Cl4 [3.213 Å, 173.7°] interaction to the solvent ethanol) while the others [Cl2 and Cl3] only in the change-assisted hydrogen bonding (Figure 6a).

The asymmetric unit thus forms a XB and HB assisted cyclic structure, where two of Cl^−^ anions are bonded between the four iodine donors by forming nearly linear I1···Cl1···I15 (~175°) and I8···Cl4···I22 (~172°) halogen bonds. Two remaining Cl^−^ anions are hydrogen bonded through N13–H···Cl2···H–N6 (~102°) and N18–H···Cl3···H–N25 (~103°) interactions.

In crystal lattice these structures forms planar layers, which are packed on top of each other as in Figure 6b shows. Additional information about the relative strength of the halogen bonding in halopyridinium halides was evaluated by analysing the corresponding bromide and chloride salts. Substituting iodine with bromine or chlorine, reducing the polarizability of halogen substituent, was envisaged to show a gradual elongation of X···Cl^−^ contact distance [24–26]. Thus the structures 3-IPyBnCl (**9**) and 3-IPyHCl (**10**) were compared with the previously published 3-BrPyHCl (**11**, CIHBAX) [41] and 3-ClPyHCl (**12**, VOQMUJ) [42]. Relevant covalent bond, hydrogen bond and halogen bond lengths and angles are depicted in Table 4.

The salts 3-IPyHCl (**10**), 3-BrPyHCl (**11**) and 3-ClPyHCl (**12**) form a series of halopyridinium chlorides where only the size and polarizability of the halogen atom differ. The charge-assisted hydrogen bond network remains the same, but the halogen bond interaction strength should vary. Surprisingly, the X-ray structures of **10**–**12** are not polymorphs, in contrast what would be predicted from the series of haloanilinium chlorides (**2**, **3**) or haloanilinium dihydrogenphosphates (**6**, **7** and **8**). The salts **11** and **12** crystallize in a triclinic space group *P*-1 and unit cell volumes are nearly equal, but the cell parameters are clearly different, viz. *a* = 5.7350(6) Å, *b* = 7.1716(6), *c* = 8.4760(8) Å, *α* = 73.365(6)°, *β* = 77.773(6)°, *γ* = 83.912(6)° for **11** and *a* = 4.7691(10) Å, *b* = 7. 744(2) Å, *c* = 9.153(2) Å, *α* = 84.26(3)°, *β* = 76.91(3)°, *γ* = 86.06(3)° for **12**, thus these are isostructural. Hydrogen bond lengths and angles are comparable and therefore the differences in the cell parameters could be explained by differences in halogen bond distances, angles and the size of the halogen atom (Table 4).

### Halogen and hydrogen bonding in 3-IPyH-5-NIPA (13)

**Figure 7:**
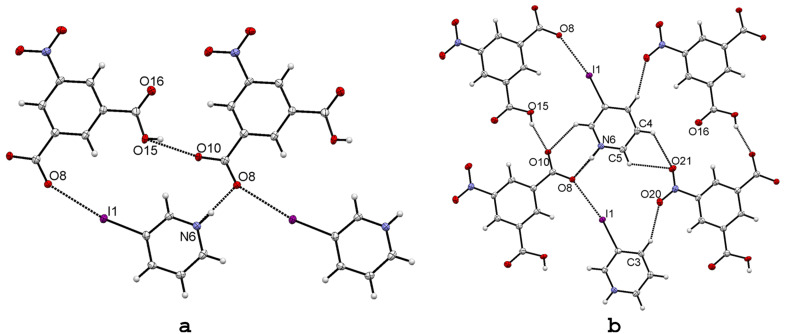
X-ray structure of 3-IPyH-5-NIPA (**13**) with selected numbering scheme of the asymmetric unit (a). A selected part of the packing is shown on (b). Thermal ellipsoids are drawn at the 50% probability level. Hydrogen and halogen bonds are shown in dotted lines. The contact distances and angles are; [I1···O8] 2.999(2) Å and 170.0(1)°, [N6–H···O8 x_1, y+1, z_] 2.625(3) Å and 175(3)°, [O15–H···O10] 2.586(2) Å and 154(4)°.

To compare the spherical and tetrahedral anions with varying HB strength to a planar strong HB anion, we selected 5-nitroisophthalic acid as a strong planar HB donor and studied its effect on the XB interactions. The 5-nitroisophthalate differs from halides by providing two hydrogen bond donors with different HB strength, thus the ratio [D^+^–H···] : [C–I···] interaction sites in **13** is 2 : 1. In addition to the disturbance in the HB interactions, the nitro groups were expected to form competing halogen bond acceptor sites as demonstrated in our previous study on co-crystals of 1-iodo-3,5-dinitrobenzene and DABCO (1,4-diazabicyclo[2.2.2]octane) [33]. Thus, we prepared X-ray quality crystals of **13** from an ethyl acetate solution of 3-iodopyridine and 5-nitroisophthalic acid in 2 : 1 molar ratio. Despite the stoichiometry employed in the crystallization experiments only 1 : 1 salts was obtained (Figure 7). A strong halogen bond is formed between the iodine atom and one of the carboxylate’s oxygens, **R** = 0.86 for I1···O8 XB distance. The planar 5-nitroisophthalate anion as a bridging moiety shows in addition to strong HB and moderately strong XB also C–H···O hydrogen bonds (Figure 7b).

## Conclusion

Among the haloanilinium salts **1**–**5** the C–I···Cl^−^ type halogen bonding occurred only in **1**, where despite the presence of three strong N–H···Cl^−^ hydrogen bonds, it had a significant effect on the observed supramolecular architecture. The gradual diminishing of the C–X···Cl^−^ interaction upon changing the identity of the halogen substituent caused clearly visible changes to occur. The absence of halogen bonding contribution in structures **2** and **3** rendered them isomorphic, while the fluorine analogue **4** had a completely different structure with weak C–F···H interactions. The corresponding bromide **5** had remarkable similarities with the chloride **1** in the charge-assisted hydrogen bonding network, yet due to the weaker halogen bonding its role in the intermolecular interactions was not easily established. The occurrence of strong hydrogen bonding, as in the isomorphic haloanilinium dihydrogenphosphates **6**–**8**, limits the role of the halogen bond, which in these cases does not affect the supramolecular architecture. From these examples it seems apparent that only a strong type of halogen bond could successfully compete with strong hydrogen bonds. This is confirmed by the halopyridinium salts **9**–**13** which clearly represented the strongest halogen bonding in the studied series. While *N*-benzyl-3-iodopyridinium chloride (**9**) can be considered as a reference system where only halogen bonded existed, structures **10**–**13** manifested supramolecular architectures where simultaneous strong halogen and hydrogen bonding co-existed. They display interesting structural and crystal lattice variations from cyclic to planar XB–HB sheet structure in **13**, showing that the balance between HB and XB interactions indeed determines the solid state architectures in these systems.

## Supporting Information

File 1 Experimental procedures and crystallographic data tables

File 2 CIF data for compounds **1**, **5**, **6**, **8**, **9**, **10** and **13**
