# Supplementary material for: The subtle balance of weak supramolecular interactions: The hierarchy of halogen and hydrogen bonds in haloanilinium and halopyridinium salts
Source: Beilstein J Org Chem. 2010 Jan 15;6:4. doi: 10.3762/bjoc.6.4 (PMC2870930; doi:10.3762/bjoc.6.4)
Supplement: File 1 — Experimental procedures and crystallographic data tables [file Beilstein_J_Org_Chem-06-04-s001.pdf]

Experimental procedures and crystallographic data tables

Supporting Information for

The subtle balance of weak supramolecular interactions: The hierarchy of halogen and hydrogen bonds in haloanilinium and halopyridinium salts

Kari Raatikainen, Massimo Cametti and Kari Rissanen*

Address: Nanoscience Center, Department of Chemistry, University of Jyväskylä, P.O. Box 35, 40014 Jyväskylä, Finland

Email: Kari Rissanen - kari.t.rissanen@jyu.fi

* Corresponding author

X-ray diffraction analyses

Suitable single crystals were selected for the measurement of structures; 4-$IPhNH_3Cl$ (**1**), 4-$IPhNH_3Br$ (**5**), 4-$IPhNH_3H_2PO_4$ (**6**), 4-$ClPhNH_3H_2PO_4$ (**8**), 3-IPyBnCl (**9**), 3-IPyHCl (**10**) and 3-IPyH-5NIPA (3-iodopyridinium 5-nitroisophthalate, **13**). Analyses were performed using Bruker Kappa Apex II diffractometer with graphite-monochromatized Mo-K_α (λ = 0.71073 Å) radiation at 123 K. Collect software [1] was used for the data measurement and DENZO-SMN [2] for the processing. The structures were solved by direct methods with SIR97 [3] and refined by full-matrix least-squares methods with WinGX-software [4], which utilizes the SHELXL-97 module [5]. An empirical absorption correction was applied to

the collected reflections [6]. All C–H hydrogen positions were calculated using a riding atom model and N–H and O–H hydrogens were located from the electron density map after anisotropic refinement of non-hydrogen atoms, with refined torsion angle. Crystallographic data for **1**, **5**, **6**, **8–10** and **13** is reported in Table 1S.

Table 1S: Crystallographic data for structures **1**, **5**, **6**, **8**–**10** and **13**.

	**1**	**5**	**6**	**8**
Formula	$C_6H_7NI{\cdot}Cl$	$C_6H_7NI{\cdot}Br$	$C_6H_7NI{\cdot}H_2PO_4$	$C_6H_7NI{\cdot}H_2PO_4$
FW (g/mol)	255.48	299.93	317.01	225.56
Crystal system	Orthorhombic	Triclinic	Orthorhombic	Orthorhombic
Space group	$P2_1ab$	*P*-1	*Pcab*	*Pcab*
a (Å)	8.2800 (3)	10.0650(4)	7.1190(10)	7.8654(2)
b (Å)	9.8354 (5)	10.9183(3)	9.8242(2)	9.6441(3)
c (Å)	10.7554 (4)	12.6269(4)	25.8681(5)	25.0515(4)
α (º)	90	100.866(2)	90	90
β (º)	90	106.743(2)	90	90
γ (º)	90	95.177(2)	90	90
Volume (Å^3)	875.89 (6)	1289.45(7)	1985.26(6)	1900.27(8)
D_{calc} (g cm^{-3})	1.937	2.318	2.121	1.577
Z	4	6	8	8
μ (mm^{-1})	3.88	8.29	3.37	0.55
F(000)	480	828	1216	928
Crystal size (mm^3)	0.25 × 0.20 × 0.15	0.40 × 0.09 × 0.09	0.40 × 0.35 × 0.25	0.25 × 0.20 × 0.20
θ max (º)	27.50	25.00	27.50	28.50
Refl. coll./unique	9794/1900	14366/4404	24333/2271	11670/2388
R(int)	0.030	0.055	0.035	0.034
Data/restr./param.	1900/1/83	4404/0/250	2271/0/121	2388/0/121
GOF on F^2	1.05	1.06	1.15	1.06
Final R indices	$R_1 = 0.018$	$R_1 = 0.054$	$R_1 = 0.023$	$R_1 = 0.036$
[I > 2σ(I)]	$wR_2 = 0.034$	$wR_2 = 0.137$	$wR_2 = 0.051$	$wR_2 = 0.09$
ΔF peak/hole(e/Å^3)	0.31/-0.40	2.21/-1.24	0.49/-0.53	0.30/-0.41

Table 1S: Crystallographic data for structures **1, 5, 6, 8–10** and **13** (continued).

	**9**	**10**	**13**
Formula	$2(C_{12}H_{11}IN)\cdot C_2H_6O\cdot H_2O\cdot 2(Cl)$	$4(C_5H_5IN)\cdot C_2H_6O\cdot 4(Cl)$	$C_8H_4NO_6\cdot C_5H_5IN$
FW (g/mol)	727.22	1011.87	416.12
Crystal system	Monoclinic	Triclinic	Triclinic
Space group	$P2_1/n$	*P*-1	*P*-1
a (Å)	8.1841(1)	8.1913(1)	8.1247(2)
b (Å)	16.9194(2)	11.9183(2)	8.4128(2)
c (Å)	20.4094(3)	17.9429(3)	11.7526(3)
α (º)	90	80.027(1)	87.951(2)
β (º)	94.60630(3)	78.217(1)	73.032(2)
γ (º)	90	76.302(1)	62.987(2)
Volume (Å^3)	2816.98(6)	1582.66(4)	680.09(3)
D_{calc} (g cm^{-3})	1.715	2.123	2.032
Z	4	2	2
μ (mm^{-1})	2.45	4.30	2.39
F(000)	1424	948	404
Crystal size (mm^3)	0.28 × 0.16 × 0.14	0.40 × 0.28 × 0.20	0.25 × 0.20 × 0.20
θ max (º)	27.50	27.50	25.00
Refl. coll./unique	39929/6478	19765/6784	7584/2322
R(int)	0.048	0.029	0.031
Data/restr./param.	6478/0/309	6784/0/318	2322/1/207
GOF on F^2	1.04	1.02	1.07
Final R indices	$R_1 = 0.033$	$R_1 = 0.033$	$R_1 = 0.023$
[I > 2σ(I)]	$wR_2 = 0.068$	$wR_2 = 0.072$	$wR_2 = 0.050$
ΔF peak/hole(e/Å^3)	1.05/-0.50	1.13/-0.91	0.59/-0.41

References

1 Hooft, R. W. *COLLECT;* Nonius BV: Delft, The Netherlands, 1998.

2 Otwinowski, Z.; Minor, W. *Methods Enzymol.* **1997**, *276* (Volume title: Macromolecular Crystallography, Part A), 307–326. doi:10.1016/S0076-6879(97)76066-X

3 Altomare, A.; Burla, M. C.; Camalli, M.; Cascarano, G. L.; Giacovazzo, C.; Guagliardi, A.; Moliterni, A. G. G.; Polidori, G.; Spagna, R. *J. Appl. Crystallogr*. **1999**, *32*, 115–119. doi:10.1107/S0021889898007717

4 Farrugia, L. J. *J. Appl. Crystallogr*. **1999**, *32*, 837–838. doi:10.1107/S0021889899006020

5 Sheldrick, G. M. *SHELXL-97 – A program for the Refinement of Crystal Structures*, Release 97-2; Universität Göttingen: Göttingen, Germany 1997.

6 Blessing, R. H. *Acta Crystallogr., Sect. A* **1995**, *51*, 33–38. doi:10.1107/S0108767394005726
